# Lifespan Increase of *Podospora anserina* by Oleic Acid Is Linked to Alterations in Energy Metabolism, Membrane Trafficking and Autophagy

**DOI:** 10.3390/cells11030519

**Published:** 2022-02-02

**Authors:** Lea Schürmanns, Andrea Hamann, Heinz D. Osiewacz

**Affiliations:** Faculty of Biosciences, Institute for Molecular Biosciences, Goethe University, Max-von-Laue-Street 9, 60438 Frankfurt, Germany; schuermanns@bio.uni-frankfurt.de (L.S.); a.hamann@bio.uni-frankfurt.de (A.H.)

**Keywords:** *Podospora anserina*, aging, autophagy, membrane trafficking, ATG24, peroxisomes, ER, mitochondria

## Abstract

The maintenance of cellular homeostasis over time is essential to avoid the degeneration of biological systems leading to aging and disease. Several interconnected pathways are active in this kind of quality control. One of them is autophagy, the vacuolar degradation of cellular components. The absence of the sorting nexin PaATG24 (SNX4 in other organisms) has been demonstrated to result in impairments in different types of autophagy and lead to a shortened lifespan. In addition, the growth rate and the size of vacuoles are strongly reduced. Here, we report how an oleic acid diet leads to longevity of the wild type and a *PaAtg24* deletion mutant (*ΔPaAtg24*). The lifespan extension is linked to altered membrane trafficking, which abrogates the observed autophagy defects in *ΔPaAtg24* by restoring vacuole size and the proper localization of SNARE protein PaSNC1. In addition, an oleic acid diet leads to an altered use of the mitochondrial respiratory chain: complex I and II are bypassed, leading to reduced reactive oxygen species (ROS) production. Overall, our study uncovers multiple effects of an oleic acid diet, which extends the lifespan of *P. anserina* and provides perspectives to explain the positive nutritional effects on human aging.

## 1. Introduction

The proper development of biological systems and the ability to adapt to changing environmental situations is strongly controlled by a complex network of molecular pathways. Impairments of these pathways lead to dysfunctions, disease and aging. We use the filamentous ascomycete *Podospora anserina* to unravel the molecular basis of organismic aging and development. Over the years, several molecular pathways have been described to play a role in the control of cellular homeostasis and aging of this well-established aging model (for a current review, see [1]). One of the identified pathways is autophagy. In aged *P. anserina* cultures, an increased number of autophagosomes is observed. In addition, the absence of the serine/threonine kinase PaATG1, which is crucial for the formation of autophagosomes, results in a lifespan that is shorter than that of the wild type [2].

During autophagy, membrane availability is important to create enough capacity for vacuole and autophagosome formation. The protein family of sorting nexins is involved in this process and plays a role in vesicle transport, membrane trafficking and protein sorting [3,4,5]. A role of sorting nexins in endosomal sorting is also described [6]. The sorting nexin SNX4 impacts autophagy. In yeast, the absence of SNX4 results in impaired pexophagy [5], nonselective autophagy and compromises autophagosome–vacuole fusion [7]. In addition, SNX4 mediates recycling of the vesicle synaptosome-associated receptor protein (v-SNARE) SNC1 [8], which is active in controlling the fusion of secretory vesicles with the plasma membrane.

In a previous study, we analyzed PaATG24, a *P. anserina* orthologue of human and yeast SNX4, and found that the deletion of *PaAtg24* leads to a significantly reduced growth rate and lifespan [9]. In addition, fertility is strongly affected. These phenotypical features go along with pronounced impairments in mitophagy, pexophagy and non-selective autophagy. The vacuoles of the mutant are significantly smaller than those of the wild type, suggesting that vacuolar membrane trafficking is impaired. Moreover, a strong age-dependent accumulation of peroxisomes was observed in the absence of PaATG24 [9]. To analyze whether or not these peroxisomes are functional, growth tests on oleate-containing medium were performed. In *P. anserina*, oleate is exclusively metabolized via the peroxisomal β-oxidation of fatty acids [10]. Surprisingly, an oleic acid diet normalizes the growth rate of *ΔPaAtg24* [9].

Here we report investigations to elucidate the beneficial effect of an oleic acid diet on aging in *P. anserina* in more detail. We show that this special diet leads to longevity of both the wild type and the *PaAtg24* deletion mutant and is linked to alterations in energy metabolism, membrane trafficking and autophagy.

## 2. Materials and Methods

### 2.1. P. anserina Strains and Cultivation

For this study, *P. anserina* wild-type strain “s” [11], as well as the mutants *ΔPaAtg24*, *mCherry-SKL*, *ΔPaAtg24/mCherry-SKL*, *ΔPaAtg24/Gfp::PaAtg8* [9], *Gfp::PaAtg8* [2], *PaSod3^H26L^::Gfp* [12] and the newly generated strains *PaSnc1::mCherry*, *ΔPaAtg24/PaSnc1::mCherry, MoMmCherry*, *ΔPaAtg24/MoMmCherry*, *ERmCherry*, *ΔPaAtg24/ERmCherry*, *ΔPaAtg37*, *ΔPaAtg37/mCherry-SKL*, *ΔPaAtg37/PaSod3^H26L^::Gfp* and *ΔPaAtg37/PaSod3^H26L^::Gfp-SKL* were used. If not otherwise stated, strains were cultivated on M2 agar plates at 27 °C under constant light conditions [13]. Ascospore germination was induced on BMM plates containing 60 mM ammonium acetate (Merck, Darmstadt, Germany; 1116.1000) for 2 days at 27 °C in the dark. All strains used in this study were derived from monokaryotic ascospores. For lifespan and growth rate analysis, strains were grown on solid M2 medium [13] or for oleic acid diet, on M2O medium, in which dextrin was replaced by 0.2% Tween^®^ 40 (Sigma-Aldrich, St. Louis, MO, USA; P1504) and 0.05% oleic acid (Sigma-Aldrich, St. Louis, MO, USA; O1008). For the extraction of total protein extracts, mycelia from *P. anserina* strains were cultivated for two days on M2 agar plates covered with cellophane foil at 27 °C under constant light, followed by cultivation in CM liquid medium (with 1% glucose) [13] for 3 days at 27 °C under constant light and shaking. For experiments with oleic acid, M2O agar plates and CMO liquid medium (with 0.2% Tween^®^ 40 and 0.05% oleic acid instead of glucose) were used. For the isolation of secreted proteins, strains were grown on M2 or M2O agar plates covered with cellophane foil for 3 days at 27 °C under constant light, followed by an incubation in Fernbach flasks with 150 mL liquid media (CM or CMO) for 4 days at 27 °C under constant light. For fluorescence microscopic analyses, *P. anserina* strains were cultivated on glass slides with a central depression containing 130 µL M2 or M2O agar medium for 1–2 days at 27 °C under constant light.

### 2.2. Cloning Procedure and Generation of P. anserina Mutants

To generate a plasmid that encodes N-terminal mCHERRY-tagged PaSNC1 (UniProt B2AT44), a two-step procedure was used. First, two inserts were generated by PCR. Insert 1 comprises the *PaSnc1* promoter and was amplified with oligonucleotides Snc1P1 (CGGGTACCGTGGCACATATTGGAC, KpnI site underlined; Biomers, Ulm, Germany) and Snc1P2 (ATGGATGACGACTGTCGCTGGC, 5′ phosphate; Biomers, Ulm, Germany) with genomic DNA as template. The amplification product was subsequently digested with KpnI (Thermo Scientific, Waltham, MA, USA; ER0521). Insert 2 covers the *mCherry* gene and was amplified with oligonucleotides mCherry-P (ATGGTGAGCAAGGGCGAGGA, 5′ phosphate; Biomers, Ulm, Germany) and mCherryR-ClaI (GCCCATCGATCTTGTACAGCTCGTCCATGC, ClaI site underlined; Biomers, Ulm, Germany) from template pLS1 [14] and finally digested with ClaI (Thermo Scientific, Waltham, MA, USA; ER0141). To generate plasmid pSnc1-A, insert 1 and 2 were cloned in the ClaI/KpnI digested backbone of plasmid pKO6 [2]. Second, the *PaSnc1* promoter region together with the *mCherry* gene was amplified from pSnc1-A (Snc1P1/mCherry-AH4) and digested with KpnI. In parallel, the *PaSnc1* open reading frame (ORF) and terminator region were amplified with Snc1-1 (ATGTCTTCCGAGCCGTATG, 5′ phosphate; Biomers, Ulm, Germany) and Snc1-2 (GGATCGATATGTGAAAGCGTCGTGTC, ClaI site underlined; Biomers, Ulm, Germany). As a template, genomic DNA was used and the resulting amplification product was digested with ClaI. Both inserts were cloned into KpnI/ClaI-digested pKO6 to generate plasmid pPaSnc1mCherry. This plasmid contains the *PaSnc1* gene controlled by its own promoter and terminator region and a 5′ fusion with the *mCherry* ORF. Finally, 10 µg of this plasmid was transformed into spheroplasts of wild-type strain “s” as described earlier [13]. For subsequent studies, a transformant with a single ectopic integration of the plasmid (identified by Southern blot analysis) was used.

To obtain plasmid pERmCherry, a DNA fragment was generated containing the *mCherry* gene with endoplasmatic reticulum (ER) signaling sequence and ER retention signal from *PaNoxD* [15] (PaNOXD: Uniprot B2A8S8) at the 5′ end to direct the *mCherry* gene product to the ER. Therefore, primers were used that add the corresponding sequences to the *mCherry* ORF (from plasmid pLS1): ER_Sigfor2 (CAAGGATCCGCCACCATGGGAAAGCTTATCAAGAACCACTGGGCGAGGCTCATCATCCTGGCCTCGGCAACTTACCAGATTGCCGCTGCGATAGAAATGGTGAGCAAGGGCGAGGAGGATAAC, BamHI site underlined; Biomers, Ulm, Germany) and ER_Retrev (CCGCCTCTAGACTAGAGCTCGTCCTTGTACAGCTCGTCCATGC; XbaI site underlined; Biomers, Ulm, Germany). The PCR product was digested with BamHI and XbaI (Thermo Scientific, Waltham, MA, USA; ER0051, ER0681) and inserted into the BamHI/XbaI backbone of pExMtterhph [16]. Similarly, plasmid pMOMmCherry was generated using primers MOMfor (CAGGATCCGCCACCATGAAGAGCTTCATTAC, BamHI site underlined; Biomers, Ulm, Germany) and ScTOM70rev (TCCAGCGCTAGCGCGGCC, 5′ phosphate; Biomers, Ulm, Germany) using pFR5637 as a template [17]. Primer ScTOM70rev introduces the sequence encoding the transmembrane domain of the yeast outer membrane protein TOM70 (Uniprot P07213), which allows the direction of the *mCherry* gene product to the mitochondrial outer membrane. The PCR product was digested with BamHI. In parallel, the *mCherry* gene was amplified with mCherry-P (ATGGTGAGCAAGGGCGAGGA, 5′ phosphate; Biomers, Ulm, Germany) and MOMTFPrev (GCCCTCTAGATTACTTGTACAGCTCGTCCATGC, XbaI site underlined; Biomers, Ulm, Germany) using pLS1 as a template [14] and subsequently digested with XbaI. Both inserts were cloned into the BamHI/XbaI backbone of pExMtterhph. In pERmCherry and pMOMmCherry, the *mCherry* gene is under the control of the metallothionein promoter and terminator region [18], allowing constitutive gene expression. After transformation of these plasmids in spheroplasts of wild-type strain “s”, transformants with single ectopic integration of the respective plasmid were identified and used for the microscopic visualization of ER and mitochondria. In addition, ERmCHERRY strains were used to measure ER-phagy flux by determining the ratio of free mCHERRY/(ERmCHERRY + free mCHERRY) by western blot analyses.

Construction of *ΔPaAtg37* was performed as previously described by [19]. To create a strain without PaATG37 (Uniprot B2AZA3), first deletion plasmid pATG37KO1 was constructed. Therefore, the flanking regions of *PaAtg37* were amplified from a *PaAtg37*-containing cosmid of a representative cosmid library [20] with the oligonucleotides Atg37KO1 (CGCTCGAGGCCATGTCAAACATCATGGACAG, XhoI site underlined; Biomers, Ulm, Germany) and Atg37KO2 (CCGCCGCGAAGCTTGTTGAACTATTATACTTGCC, HindIII site underlined; Biomers, Ulm, Germany) for the 5′-flank of *PaAtg37* and Atg37KO3 (TAACTGCAGGGAGGGGGGTTTCAAGAG, PstI site underlined; Biomers, Ulm, Germany) and Atg37KO4 (TTGCGGCCGCAGGAGAATCTTTTGCTGG, NotI site underlined; Biomers, Ulm, Germany) for the 3′-flank of *PaAtg37*. The fragments were digested with XhoI and HindIII or PstI and NotI (Thermo Scientific, Waltham, MA, USA; ER0691, ER0501, ER0611, ER0591), respectively. Both inserts were cloned in the backbone of pKO7 [21], which had previously been digested with all four enzymes. Next, 10 µg of this plasmid was transformed into spheroplasts of the *ΔPaKu70* strain [19], as described earlier [13], and the resulting transformants were selected on hygromycin B-containing medium (Calbiochem, San-Diego, CA, USA, 400051). The final *ΔPaAtg37* strain containing the wild-type *PaKu70* gene was subsequently selected from the progeny of a cross of the selected transformants and the wild type. The constructed *ΔPaAtg37* strain was verified by Southern blot analysis.

All transgenic strains are in the genetic background of the wild-type strain “s”. For double mutant generation, the single mutant strains were crossed with each other and progenies used for further experiments were selected containing both mutations.

### 2.3. Growth Rate and Lifespan Determination

Lifespan and growth rate analysis were performed on solid M2 or M2O medium according to [13]. Briefly, small mycelium pieces of two-day-old cultures were placed on agar medium and incubated at 27 °C under constant light. Growth was recorded every day until it stopped. For special growth tests, rotenone (inhibitor of complex I, Sigma-Aldrich, St. Louis, MO, USA; R8875) or malonate (inhibitor of complex II; Merck, Darmstadt, Germany; 800380) or both was added to the medium after sterilization. The lifespan of *P. anserina* is defined as the time period in days (d) of hyphal growth until growth stop, while the growth rate is defined as the measured growth (cm) per day.

### 2.4. Southern Blot Analyses

The isolation of DNA from *P. anserina* was performed according to the protocol of Lecellier and Silar [22]. DNA digestion, gel electrophoresis and Southern blotting were carried out using standard protocols. Southern blot hybridization and detection was carried out as described in the manufacturer’s protocol with digoxigenin-labeled hybridization probes (DIG DNA Labeling and Detection Kit; Roche Applied Science, Mannheim, Germany; 11175033910). The *PaAtg24*-, *PaAtg37*, *hph*- and *mCherry*-specific probes were generated from plasmids containing the respective genes. The *PaAtg24* gene was detected with an NcoI/XhoI-digested (Thermo Scientific, Waltham, MA, USA; ER0571, ER0691) fragment encompassing a part of the ORF of *PaAtg24*. The hygromycin resistance gene *hph* was detected with a NcoI/ClaI-digested fragment from the pKO7 plasmid [21]. A specific *mCherry* fragment was amplified from the pLS1 [14] plasmid using the oligonucleotides mCherry-P and mCherryR-ClaI. The *PaAtg37*-specific probe contains a *PaAtg37*-fragment amplified with oligonucleotides Atg37-1 (GGAGCAAGACAAGTGGGATG; Biomers, Ulm, Germany) and Atg37-2 (TCCCTCAACGCGGCGATTTC; Biomers, Ulm, Germany).

### 2.5. Isolation of Total Protein Extracts

Freshly grown mycelium (250 mg) was transferred into a tube filled with glass beads (Precellys^®^ Lysing Kit, Bertin Technologies, Montigny-le-Bretonneux, France; KT0393-1-004.2) and mixed with 2 volumes of protein extraction buffer containing 1 mM EDTA (Merck, Darmstadt, Germany; 1.08418.1000), 20 mM HEPES (Serva, Heidelberg, Germany; 25245) and 5 mM DTT (Carl Roth, Karlsruhe, Germany; 6908.4). The pH value was adjusted to 7.5 with NaOH (Carl Roth, Karlsruhe, Germany; 6771.1). The mycelia were homogenized twice for 25 s and 5800 rpm in a homogenizer (Precellys 24 homogenizer; Bertin Technologies, Montigny-le-Bretonneux, France), with a 10 s rest between the steps, followed by a final centrifugation for 5 min at 9300× *g* at 4 °C. The supernatant was used in western blot analyses.

### 2.6. Isolation of Secreted Proteins

For the isolation of the secreted proteins, the protocol of Zintel and colleagues was followed [23]. Briefly, the filtered liquid growth medium was enriched by filter tubes (Amicon^®^ Ultra-15, Ultracel-3k; Merck Millipore, Burlington, MA, USA; MPUFC900324). Supernatants were mixed with 1:100 PIC (Protease Inhibitor Cocktail Set IV; Calbiochem, San Diego, CA, USA; 539136). The secreted proteins were precipitated by adding 2 volumes of ethanol and 2 volumes of acetone for 2 days at −20 °C. After centrifugation (30 min, at 4000× *g*), 1 mL desalinization solution (water:ethanol:acetone 2:1:1) was added to the pellet. After 1 min of mixing, the secreted proteins were incubated overnight at −20 °C. This desalinization procedure was repeated twice. The pellets were dried and resolved with 50 μL protein extraction puffer at room temperature.

### 2.7. Western Blot Analysis

The protein amount of the total protein extracts or secreted protein samples was measured spectrophotometrically according to a protocol from Bradford [24] with ROTI^®^Nanoquant (Carl Roth, Karlsruhe, Germany; K880.1) at 450 and 590 nm using bovine serum albumin (BSA; Sigma-Aldrich, St. Louis, MO, USA; A6003) as reference. Then, 100 µg of total protein extracts or 10 µg of secreted protein samples were separated by 2-phase SDS-PAGE (12% separating gels) according to the standard protocol [25]. Subsequently, protein transfer to PVDF membranes was performed, according to the manufacturer’s specifications, using the Trans-Blot^®^ Turbo™ transfer system (BIO-RAD, Hercules, CA, USA). Afterwards, blocking of the membrane, antibody incubation and washing steps were performed according to the Odyssey^®^ handbook for western blot analysis (LI-COR Biosciences, Bad Homburg, Germany). The following primary antibodies were used: anti-GFP (mouse; dilution 1:10,000; Sigma-Aldrich, St. Louis, MO, USA; G6795), anti-mCHERRY (mouse; dilution 1:3000; Sigma-Aldrich, St. Louis, MO, USA; SAB2702286), anti-PaCATB (rabbit; dilution 1:10,000; New England Peptide, Gardner, MA, USA), raised against a PaCATB-specific synthetic peptide (Ac-CRYLGRFPVDEGAE-OH) and anti-PaIDI1 (rabbit; dilution 1:10,000; New England Peptide, Gardner, MA, USA), raised against a PaIDI-1-specific synthetic peptide (Ac-GARSAEPYVLRDSISYRC-OH). Afterwards, IR Dye^®^ 680RD anti-rabbit (goat; dilution 1:15,000; LI-COR Biosciences, Bad Homburg, Germany; 926-68071), IRDye^®^ 800CW anti-mouse (goat; dilution 1:15,000; LI-COR Biosciences, Bad Homburg, Germany; 926-32210) or IRDye^®^ 680RD anti-mouse (goat; dilution 1:15,000; LI-COR Biosciences, Bad Homburg, Germany; 926-68070) were used as conjugated secondary antibodies. For detection, the Odyssey^®^ Fc imaging system (LIC-OR Biosciences, Bad Homburg, Germany) was used. Densitometric quantification was performed with the manufacturer’s software Image Studio™. The Coomassie-stained gel was used as a loading control.

### 2.8. Measuring the Rate of Peroxisomal β-Oxidation of Fatty Acids

The rate of peroxisomal β-oxidation of fatty acid was performed according to Lazarow (1981) [26]. During the third step of this process, NAD is reduced to NADH, which is measured spectrophotometrically at 340 nm. The following ingredients were mixed in a cuvette: 945 µL TRIS-HCl buffer (50 mM; pH 8; Carl Roth, Karlsruhe, Germany; 4855.3), 10 µL NAD (20 mM; Sigma-Aldrich, St. Louis, MO, USA; N1511), 10 µL FAD (1 mM; Sigma-Aldrich, St. Louis, MO, USA; F8384), 10 µL CoA (10 mM; Sigma-Aldrich, St. Louis, MO, USA; C3144), 5 µL BSA (15 mg/mL), 5 µL Triton X-100 (20 mg/mL; Carl Roth, Karlsruhe, Germany; 3051.3), 3 µL DTT (0.33 M) and 100 µg total protein extract (in 10 µL). To start the reaction, 2 µL palmitoyl-CoA (5 mM; Sigma-Aldrich, St. Louis, MO, USA; P9716), a peroxisome specific substrate, was added. Every sample was measured three times (technical replicates) every 15 s for 5 min. The measured values of absorbance versus time are converted to the rate of peroxisomal β-oxidation of fatty acid.

### 2.9. In-Gel Peroxidase and Catalase Activity Assay

The method was basically adapted from a previously published protocol [27]. First, 30 µg of total protein extract was loaded on three identical 10% native polyacrylamide gels. Afterwards, electrophoresis for 16 h at 70 V and 4 °C was performed. Gel 1 was stained with Coomassie as a loading control. Gel 2 was used for peroxidase activity staining: the gel was washed three times for 10 min in PBS (pH 7.4) and then incubated in 30 mL DAB solution (30 mg diaminobenzidine; Sigma-Aldrich, St. Louis, MO, USA; D8001) dissolved in 30 mL PBS (pH 7.4) and 3 µL 30% hydrogen peroxide (Carl Roth, Karlsruhe, Germany; 8070.2) for 20 min under shaking. To stop the reaction, the gel was washed in H_2_O twice. Gel 3 was used for catalase activity staining: the gel was washed with H_2_O three times (10 min each) and subsequently incubated in 30 mL of H_2_O with 3 µL of 30% hydrogen peroxide under shaking in the dark. The gel was washed with H_2_O twice and was incubated for 10 min on a light table with 2 g FeCl_3_ × 6 H_2_O (Sigma-Aldrich, St. Louis, MO, USA; P742.1) in 10 mL H_2_O and 1.2 g K_3_[Fe(CN)_6_] (Carl Roth, Karlsruhe, Germany; 244023) in 10 mL H_2_O. Both solutions were mixed and immediately used. Afterward, the gel was washed with H_2_O and documented by scanning.

### 2.10. Hydrogen Peroxide Emission Measurements

The hydrogen peroxide emission was measured in two ways: qualitative measurements were performed by monitoring the oxidation of DAB according to the protocol of Munkres [28]. *P. anserina* strains were cultivated for 4 days on M2 or M2O agar plates at 27 °C in the dark. Subsequently, the plates were floated with staining solution, containing 2.5 mM DAB in 100 mM TRIS-HCl buffer (pH 6.9), and incubated for 30 min in the dark at 27 °C. Afterwards, the solution was removed and the plates were incubated for an additional 3 h under the same conditions. The plates were immediately documented by scanning.

Quantitative measurements of hydrogen peroxide emission were performed according to Kowald et al. [29]. Briefly, the *P. anserina* strains were cultivated on M2 or M2O agar plates for 4 days at 27 °C under constant light. Subsequently, round pieces of mycelium were punched out of the growth front using an open 0.2 mL microcentrifuge tube and transferred to a 96-well microwell plate. The mycelium was overlaid with 200 µL of staining solution (100 mM TRIS (pH 6.9), 0.02 mg/mL horseradish peroxidase (Sigma-Aldrich St. Louis, MO, USA; P8250) and 2.5 mM DAB. After incubation at 27 °C for 3 h in darkness, 100 µL of the supernatant was transferred into a fresh 96-well microwell plate and the absorption was measured at 471 nm in a microplate reader (Safire 2, Tecan, Maennedorf, Switzerland). For each strain, at least three different monokaryotic ascospore isolates (biological replicates) were used and three pieces of mycelium (technical replicates) from each isolate were measured. The dry weight of the mycelium was used to normalize the absorption values. Therefore, pieces of mycelium were boiled in hot water for 90 sec to remove the agar and then air-dried for 3 days.

### 2.11. Fluorescence Microscopy

Microscopic analyses were performed with the Zeiss Cell Observer SD, a confocal spinning disc microscope, and a 63x/1.4 oil objective lens (Carl Zeiss Microscopy, Jena, Germany) using 488 nm-, 506 nm-, 587 nm- and 592 nm laser lines. For mitochondrial membrane potential analysis, TMRM staining solution (1 µM tetramethylrhodamine methyl ester perchlorate; Sigma-Aldrich, St. Louis, MO, USA; T5428; dissolved in H_2_O) was incubated for 30 min. For the staining of lipid droplets, freshly grown mycelium was incubated with LipidSpot™ 488 (Biotium, Fremont, CA; 70065) for 15 min. For the analysis of vacuoles, freshly grown mycelium was treated for 5 h with 2 μg/mL FM™ 4-64 Dye (Invitrogen, Waltham, MA, USA; T13320) before microscopic analysis. For image processing, the Zeiss microscopy software ZEN 2.5 (blue edition) was used. The size and distribution of vacuoles, as well as the intensity of TMRM, were measured manually using ZEN software.

### 2.12. Statistical Analysis

For statistical analysis, the two-tailed *t*-test was used. The threshold for statistical significance was set at a minimum of *p* > 0.05 (not significant: n. s.). * = *p* ≤ 0.05; ** = *p* ≤ 0.01; *** = *p* ≤ 0.001. All shown error bars correspond to the respective standard deviation.

## 3. Results and Discussion

### 3.1. Oleic Acid Diet Rescues Shortened Lifespan and Impaired Peroxisomal Functionality of ΔPaAtg24

**Figure 1:**
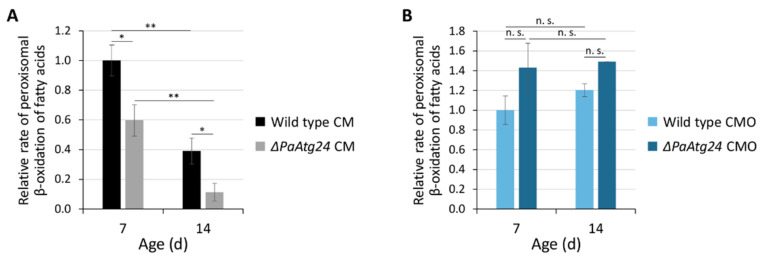
Oleic acid diet rescues peroxisomal functionality of *ΔPaAtg24*. Measurements of peroxisomal β-oxidation rate of fatty acids in 100 µg total protein extract from 7- and 14-day-old wild type and *ΔPaAtg24*, grown in (**A**) standard medium (CM) or (**B**) oleic acid-containing medium (CMO). Data represent mean ± SD (n = 3). Samples were statistically analyzed with two-tailed *t*-test (n. s. = *p* > 0.05; * = *p* ≤ 0.05; ** = *p* ≤ 0.01). For data point distribution, see Figure S1.

In a recent study, we reported that the deletion of *PaAtg24*, a gene coding for a sorting nexin, leads to a significant reduction of the growth rate and lifespan in *P. anserina* on standard growth medium [9]. In addition, fertility is strongly affected. These characteristics go along with pronounced impairments in mitophagy, pexophagy and non-selective autophagy. At the cellular level, the vacuoles of the mutant are significantly smaller than those of the wild type, suggesting that vacuolar membrane trafficking is affected. Moreover, an accumulation of peroxisomes was observed in the absence of PaATG24 in 7-day-old cultures, which increases during aging [1,9]. To analyze how far these peroxisomes are functional, we determined peroxisomal activity. Therefore, the rate of peroxisomal β-oxidation of fatty acids was measured spectrophotometrically. For this analysis, strains were grown in liquid standard medium containing glucose monohydrate (CM) or in medium containing oleic acid as the sole carbon source (CMO), which is exclusively metabolized via peroxisomal β-oxidation in *P. anserina* [10]. The measurements unraveled a significant reduction of peroxisomal β-oxidation activity during aging in the wild type and in *ΔPaAtg24* under standard growth conditions (Figure 1A). In the absence of PaATG24, the effect is even more pronounced and appears to be the result of the inability to remove the damaged, non-functional peroxisomes that accumulate during aging as a result of impairments in degradation by pexophagy and general autophagy in *ΔPaAtg24* [9]. This explains why, despite an increase of peroxisome abundance, peroxisomal activity is reduced in the mutant. Significantly, the oleic acid diet somehow “heals” the PaATG24- and age-related impairments in peroxisomal activity (Figure 1B). Although the measured activities in *ΔPaAtg24* appear to be even higher than in the wild type, statistical analysis reveals no significant differences. However, and most strikingly, the peroxisomal activity of *ΔPaAtg24*, which is lower on glucose-containing medium than that of the wild type, is restored on oleic acid medium.

**Figure 2:**
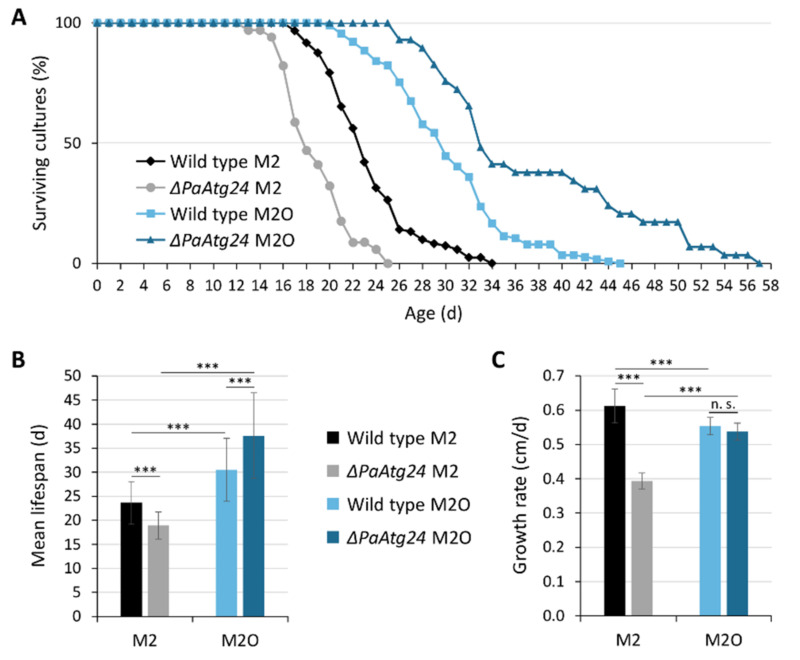
Oleic acid diet results in longevity of the wild type and of *ΔPaAtg24*. (**A**) Survival curves of *P. anserina* wild type on standard medium (M2; n = 122) and oleic acid medium (M2O; n = 115) as well as *ΔPaAtg24* on M2 (n = 36) and M2O (n = 31). (**B**) Mean lifespan and (**C**) growth rate of cultures from (**A**). Data represent mean ± SD. Samples were statistically analyzed with two-tailed *t*-test (n. s. = *p* > 0.05; *** = *p* ≤ 0.001).

Next, we performed lifespan analysis experiments to investigate whether the restoration of peroxisome activity can also revert the decrease in lifespan of *ΔPaAtg24*. Therefore, strains were grown on solid standard medium (M2) or on solid oleic acid-containing medium (M2O). Surprisingly, growth on the oleate-containing medium was found to strongly increase the lifespan of the wild type and the short-lived *ΔPaAtg24* mutant, and results in the longevity of both strains (Figure 2A,B). In addition, the growth rate of *ΔPaAtg24* on M2 is also increased by oleic acid (Figure 2C).

### 3.2. Oleic Acid Diet Affects Mitochondrial ROS Metabolism

In most of the organisms studied so far, long chain fatty acids (such as oleic acid) are converted to *trans*-Δ^2^-enoyl coenzyme A (CoA) by the enzyme acyl-CoA oxidase in the first step of peroxisomal β-oxidation. As a by-product, hydrogen peroxide (H_2_O_2_) is produced. In contrast, some fungi, such as *P. anserina*, do not possess an acyl-CoA oxidase. Instead, the enzyme acyl-CoA dehydrogenase, which uses flavin adenine dinucleotide (FAD) as co-factor [30], was described [31]. Using this enzyme, peroxisomal β-oxidation in *P. anserina* does not result in H_2_O_2_ but in FADH_2_ production. In the next steps, *trans*-Δ^2^-enoyl-CoA is converted to acyl-CoA and acetyl-CoA, resulting in the production of nicotinamide adenine dinucleotide hydrogen (NADH) as a by-product. This process can be repeated until the entire fatty acid chain is cleaved into acetyl-CoA molecules.

Peroxisome-derived FADH_2_ and NADH can be transferred to the intermembrane space of mitochondria, but not into the mitochondrial matrix, due to the impermeability of the inner mitochondrial membrane for NADH and FADH_2_. However, in *P. anserina*, an external NADH dehydrogenase transfers the electrons from external NADH in the intermembrane space to ubiquinone in the inner membrane [32]. In yeast and mammals, electrons from external FADH_2_ can be transferred to ubiquinone by mitochondrial glycerol 3-phosphate dehydrogenase [33,34]. A homologue of this protein is encoded by *P. anserina* (UniProt B2B245). Extensive use of these β-oxidation-derived reduction equivalents should reduce the necessity to use complex I and II of the mitochondrial respiratory chain. Since complex I pumps protons (H^+^) across the inner membrane, the use of this pathway should lead to a reduced mitochondrial membrane potential.

**Figure 3:**
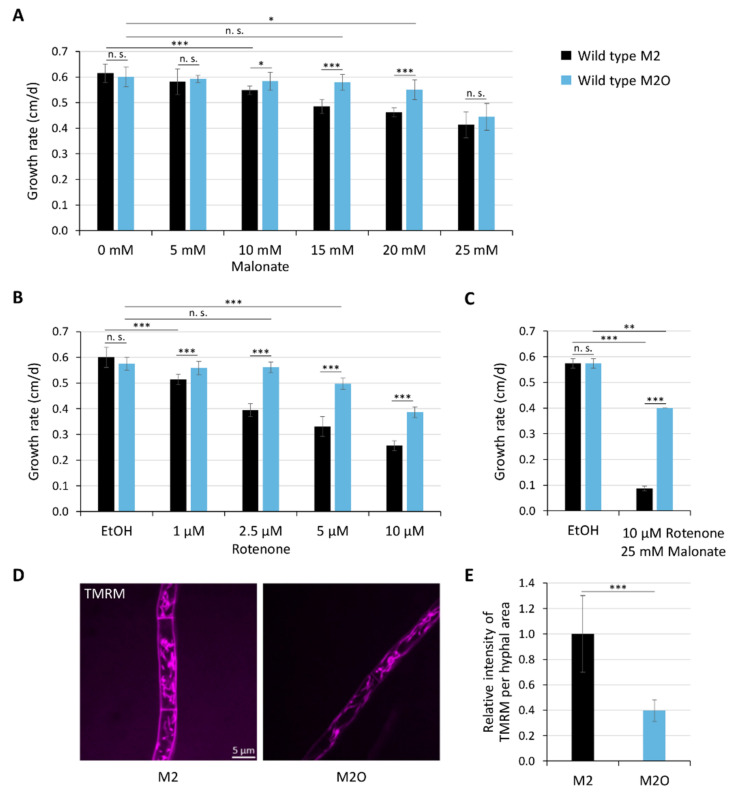
Oleic acid diet bypasses complex I and II of the mitochondrial respiratory chain. Growth tests of the wild type on standard medium (M2) and oleate-containing medium (M2O) with different concentration of complex II inhibitor malonate (**A**), complex I inhibitor rotenone (**B**) and a combination of both (**C**) were performed (n = 9). (**D**) Fluorescence microscopy of 7-day-old wild-type strains, grown on M2 or M2O medium. The samples were stained with 1 µM TMRM staining solution for 30 min. The relative intensity of the TMRM signal per hyphal area is displayed in (**E**). 2498 (M2) or 2478 µm^2^ (M2O) hyphae area was analyzed (n = 3). Data represent mean ± SD. Samples were statistically analyzed with two-tailed *t*-test (n. s. = *p* > 0.05; * = *p* ≤ 0.05; ** = *p* ≤ 0.01; *** = *p* ≤ 0.001). For data point distribution, see Figure S2.

To experimentally validate whether or not mitochondrial complexes I and II are by-passed during the metabolization of oleic acid, we performed growth tests using the wild type of *P. anserina* on medium containing rotenone (an inhibitor of complex I) and malonate (an inhibitor of complex II), respectively (Figure 3A–C). As expected, strains grown on M2 were much more dependent on functional complex I and II than those grown on M2O (growth rates on M2O at higher inhibitor concentrations are significantly better). To support these findings, we performed fluorescence microscopy with samples stained with tetramethylrhodamine methyl ester (TMRM), a positively charged fluorophore. Depending on the membrane potential, TMRM becomes differentially enriched in mitochondria. Strong signals result from a high membrane potential and an efficient uptake of the dye [35]. Growth of the wild type on M2O resulted in a 60% reduced mitochondrial membrane potential compared to that on M2 (Figure 3D,E), supporting the hypothesis that electrons from peroxisomal NADH and FADH_2_ bypass complex I and II.

**Figure 4:**
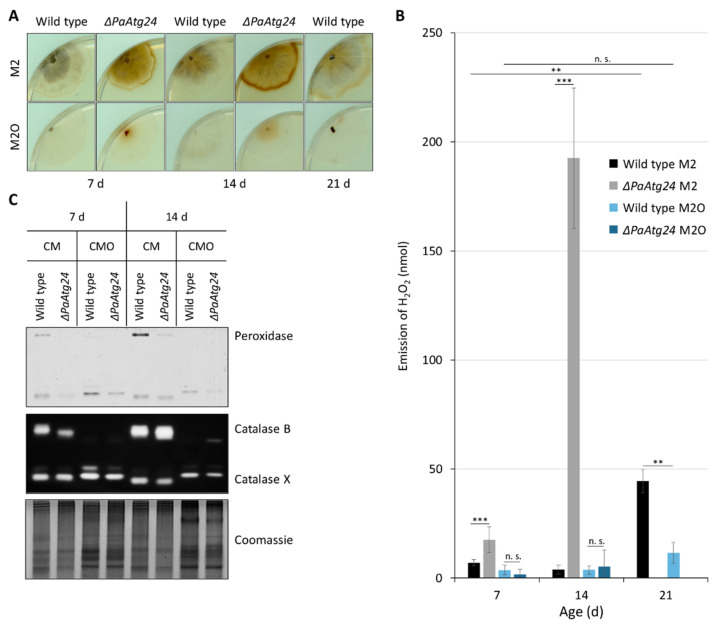
The metabolization of oleic acid results in lower hydrogen peroxide production. (**A**) Qualitative measurement of hydrogen peroxide (H_2_O_2_) release from 7-, 14- and 21-day-old wild type and 7- and 14-day-old *ΔPaAtg24* on M2 and M2O medium (n = 4) by a histochemical DAB staining. Since the mutant strain does not reach an age of 21 days on M2, there are no data available. (**B**) Quantitative measurement of H_2_O_2_ release with 7- and 14-day-old wild type and *ΔPaAtg24* on M2 and M2O medium (n = 9) as well as 21-day-old wild type (n = 3). (**C**) Peroxide and catalase activity gels from total protein extract of 7- or 14-day-old *P. anserina* wild type and *ΔPaAtg24*, grown on standard medium (CM) or oleate medium (CMO; n = 4). Data represent mean ± SD. Samples were statistically analyzed with two-tailed *t*-test (n. s. = *p* > 0.05; ** = *p* ≤ 0.01; *** = *p* ≤ 0.001). For data point distribution, see Figure S3.

The transfer of the electrons from exogenous NADH to the ubiquinone pool of the electron transport chain leads to a reduced production of ATP compared to a respiratory chain using complex I as the entrance site [32] because of the lowered membrane potential. On the other hand, fewer ROS are generated, because complex I is a major site of superoxide anion production. Subsequently, superoxide dismutase (SOD) leads to a disproportion of this ROS and the formation of H_2_O_2_, a ROS that is able to pass through the phospholipid bilayer of membranes. We determined hydrogen peroxide emission from *P. anserina* hyphae as an indirect measure of cellular ROS production. The amount of released H_2_O_2_ was measured qualitatively (Figure 4A) and was quantified (Figure 4B). 

We found that both the wild type and *ΔPaAtg24* emit increased amounts of H_2_O_2_ in old age on M2. On M2O, the H_2_O_2_ release is reduced and does not change significantly during aging. Remarkably, we found that the *PaAtg24* deletion mutant releases enormous amounts of H_2_O_2_ at age 14 d on M2. Strikingly, growth on oleic acid-containing medium neutralizes this effect (Figure 4B). To validate that indeed reduced production of H_2_O_2_ and not its increased degradation leading to the decreased H_2_O_2_ release, we determined the activities of the hydrogen peroxide-degrading enzymes. We found that the activity of the uncharacterized catalase X [23] is comparable in CM and CMO and that the activities of peroxidase and of secreted catalase B are barely detectable after growth in CMO (Figure 4C). It thus appears that the oleic acid diet results in a reduced mitochondrial H_2_O_2_ production. Accordingly, the oxygen consumption rate of cultures grown in CMO is lower than in CM (data not shown). A contribution of peroxisomes to the production of H_2_O_2_ does not occur due to the special peroxisomal β-oxidation pathway of *P. anserina*.

### 3.3. Oleic Acid Diet Abrogates the Autophagy Defect of ΔPaAtg24

**Figure 5:**
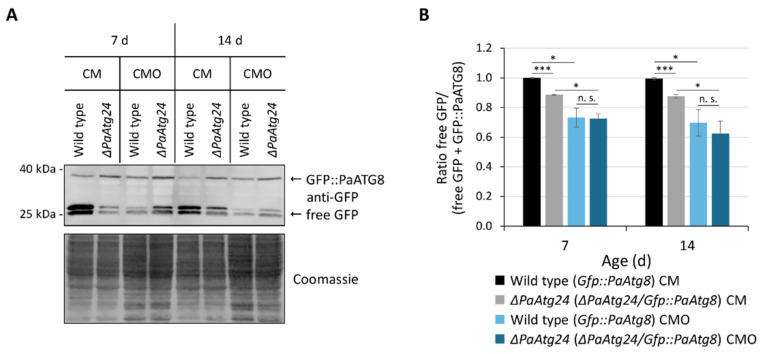
Abrogation of the *ΔPaAtg24* autophagy defect by oleic acid diet. (**A**) Western blot analysis of total protein extracts from 7- and 14-day-old *Gfp::PaAtg8* (here wild type) and *ΔPaAtg24/Gfp::PaAtg8* (here *ΔPaAtg24*), grown in liquid CM or CMO medium. The membrane was treated with an antibody against GFP. (**B**) Quantification of the ratio of free GFP to the total of free GFP and fusion protein from samples from (**A**). Data represent mean ± SD (n = 4). Samples were statistically analyzed with two-tailed *t*-test (n. s. = *p* > 0.05; * = *p* ≤ 0.05; *** = *p* ≤ 0.001). For data point distribution, see Figure S4.

Previous work in *P. anserina* uncovered connections between ROS balancing, aging and autophagy [12]. In the current study, we therefore investigated whether or not the reduction in ROS production in oleic acid-containing medium results in an altered autophagy flux. In this analysis, we used the *Gfp::PaAtg8* fusion gene to be expressed in the wild type and the *ΔPaAtg24* genetic background. As expected, we found that autophagic flux in the wild type in CMO is clearly reduced compared to growth in standard medium (Figure 5). In CM, *ΔPaAtg24* shows a reduced autophagic flux compared to that in the wild type. Most interestingly, the oleic acid diet leads to a wild-type autophagy rate in *ΔPaAtg24* in both analyzed ages (Figure 5). Since autophagy is an important quality control pathway, we assume that the mutant’s impairment in autophagy under standard conditions contributes to the high amount of emitted hydrogen peroxide of *ΔPaAtg24* at age 14 d (Figure 4B) and the reduced lifespan (Figure 2A,B).

### 3.4. Oleic Acid Diet Abolishes the Vacuole Formation Defect of ΔPaAtg24 by Normalizing Membrane Trafficking of ΔPaAtg24

In *P. anserina*, the ablation of PaATG24 was found to lead to impairments in the non-selective autophagy and selective autophagy of mitochondria and peroxisomes [9], and, as shown in this study, even in ER-phagy (Figure S5). These data suggest that PaATG24 is active in the control of basic processes that are essential for various forms of autophagy. Since in fungi the vacuole is the common compartment in which the different autophagic cargo is degraded, and since vacuoles were found to be affected in the *PaAtg24* deletion mutant [9], we surmised that the common process impaired in *ΔPaAtg24* is linked to the formation of vacuoles. This idea is supported by a recent study with yeast, in which oleic acid abrogates fragmented vacuole morphology and fusion defects in the vacuolar *ΔVac8* mutant [36].

**Figure 6:**
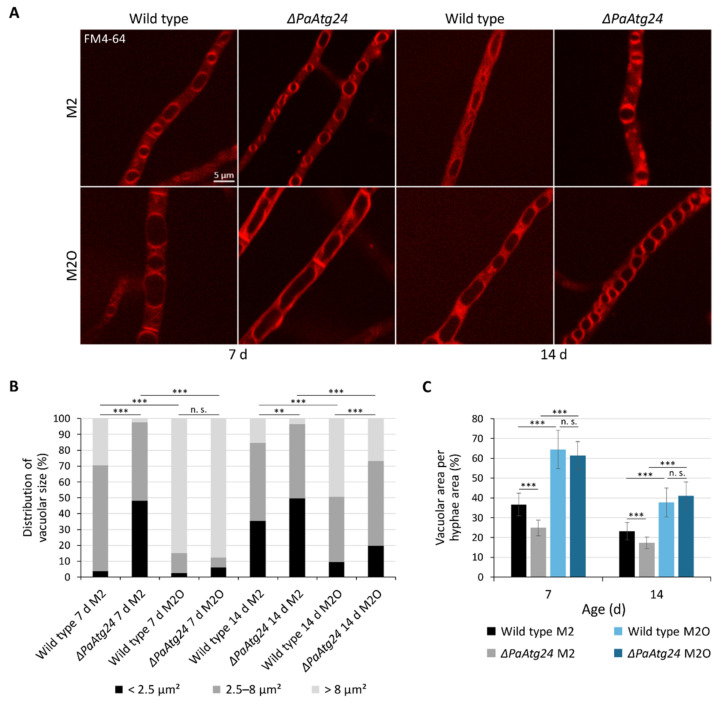
Cultivation of *P. anserina* strains on oleic acid medium leads to abrogated vacuole formation defects of *ΔPaAtg24*. (**A**) 7- and 14-day-old wild-type and *ΔPaAtg24* strains, cultivated on glass slides with M2 or M2O for 2 days followed by 5 h staining with 2 μg/mL FM4–64 for the visualization of vacuolar membranes. For analysis of the vacuolar size distribution (**B**) and the vacuolar area per area hyphae (**C**), at least 1000 µm^2^ hyphae area of each sample was analyzed. For the distribution of vacuolar size, vacuoles were sorted into three categories: smaller than 2.5 μm^2^, 2.5–8 μm^2^ and bigger than 8 μm^2^. Data represent mean ± SD (n = 3). Samples were statistically analyzed with two-tailed *t*-test (n. s. = *p* > 0.05; ** = *p* ≤ 0.01; *** = *p* ≤ 0.001).

In order to analyze a potential effect of the oleic acid diet on the formation of vacuoles in *P. anserina*, we stained mycelia of the wild type and of *ΔPaAtg24* with the lipophilic membrane dye FM4–64 and analyzed the vacuolar size and the hyphal area covered by this organelle via fluorescence microscopy on M2 and M2O (Figure 6A). FM4–64 allows the monitoring of endocytotic pathways by first staining the plasma membrane, subsequently entering the endocytotic vesicles of the endosomal pathways. Over time, FM4–64 becomes distributed throughout the full vesicular network from the plasma membrane to the vacuole [37].

We determined the vacuolar size and its distribution (Figure 6B) as well as the vacuolated area per hyphae area (Figure 6C). We found that growth on oleic acid medium results in an increase of vacuolar size and vacuolated area in 7- and 14-day-old wild-type strains (Figure 6A–C). In addition, normal vacuolar size and vacuolated area are restored by the oleic acid diet in 7-day-old samples of *ΔPaAtg24* (Figure 6A–C). In 14-day-old cultures, the vacuolar size of the mutant is smaller than that of the wild type (Figure 6B). However, the area covered by vacuoles per hyphae area is comparable to that of the wild type and significantly increased compared to the *ΔPaAtg24* sample grown on M2 at age 14 d (Figure 6C). We surmise that on oleic acid-containing medium, the wild-type-like autophagy flux of *ΔPaAtg24* (Figure 5) results from “healing” the vacuole formation defect.

**Figure 7:**
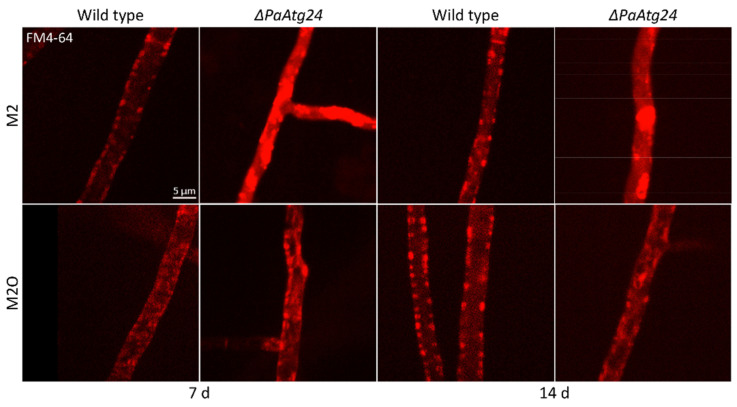
Impaired membrane trafficking in *ΔPaAtg24* is rescued by growth on oleic acid medium. Staining of 7- and 14-day-old wild type and *ΔPaAtg24* strains, grown on glass slides covered with M2 or M2O agar, respectively, for 1 day, for 5 h with 2 μg/mL FM4–64 for the visualization of membrane trafficking. For every sample, three biological replicates were analyzed.

SNX4, the yeast homologue of PaATG24, is known to play a role in membrane trafficking [7,8] and as such is linked to vacuole formation [7]. To investigate whether or not the corresponding process is affected in *ΔPaAtg24*, we used FM4–64 to compare membrane trafficking in wild type and *PaAtg24* deletion mutant. In *P. anserina*, the wild type grown on both M2 and M2O displays punctate structures localized at the plasma membrane. In the absence of PaATG24, very bright and extensive signals are observed in the cytosol on M2 medium (Figure 7), suggesting an accumulation of the dye in the cytosol and pointing to impaired endosomal sorting. The oleic acid diet appears to neutralize this effect and restores normal endosomal transport processes in *ΔPaAtg24* (Figure 7). We assume that the observed vacuole formation defect of *ΔPaAtg24* on M2 is caused by impaired membrane trafficking.

### 3.5. Oleic Acid Diet Alters Membrane Trafficking in P. anserina

To understand how the oleic acid diet normalizes impaired endosomal sorting in *ΔPaAtg24*, we examined the membrane trafficking on oleate medium in some more detail. In general, fatty acids are taken up from the environment either by diffusion through the plasma membrane or by transporters (recently reviewed in [38]). In the cell, fatty acids are stored or transported in lipid droplets. These ER-derived organelles are covered by a phospholipid monolayer, whereby the phospholipid’s hydrophilic head group points towards the cytoplasm (outwards) and the hydrophobic tail into the organelle (inwards) [39].

**Figure 8:**
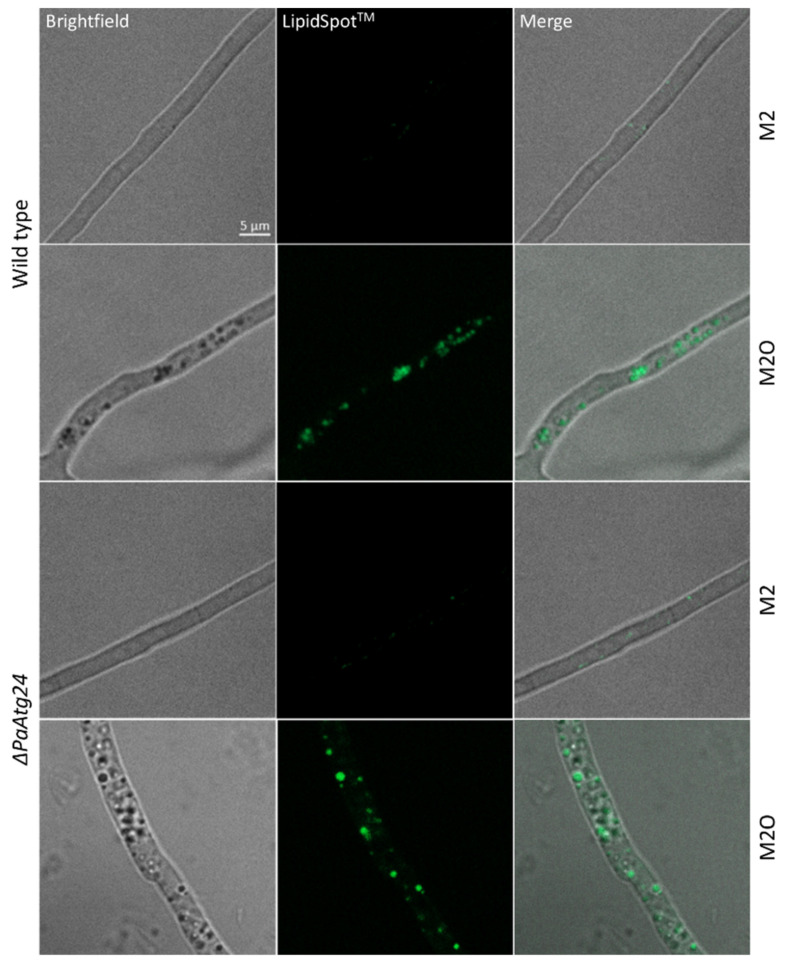
Occurrence of lipid droplets in dependence of oleic acid. Staining of 7- and 14-day-old wild type and *ΔPaAtg24* strains, cultivated on glass slides covered with M2 or M2O agar, respectively, for 1 day, for 15 min with LipidSpot™ for the visualization of lipid droplets. For every sample, three biological replicates were analyzed.

In order to investigate the role of lipid droplets in oleic acid transport, we first analyzed the occurrence of these organelles in the wild type and in *ΔPaAtg24* of two different age-stages grown on standard growth medium or on medium containing oleic acid as sole carbon source. The strains were stained with the lipid droplet dye LipidSpot™ and analyzed by fluorescence microscopy. Lipid droplets were found in both strains only on oleic acid medium (Figure 8). It appears that, in contrast to the growth on standard medium, lipid droplets are essential on oleate medium for the intracellular transport of oleic acid to peroxisomes for metabolization and survival under these conditions.

**Figure 9:**
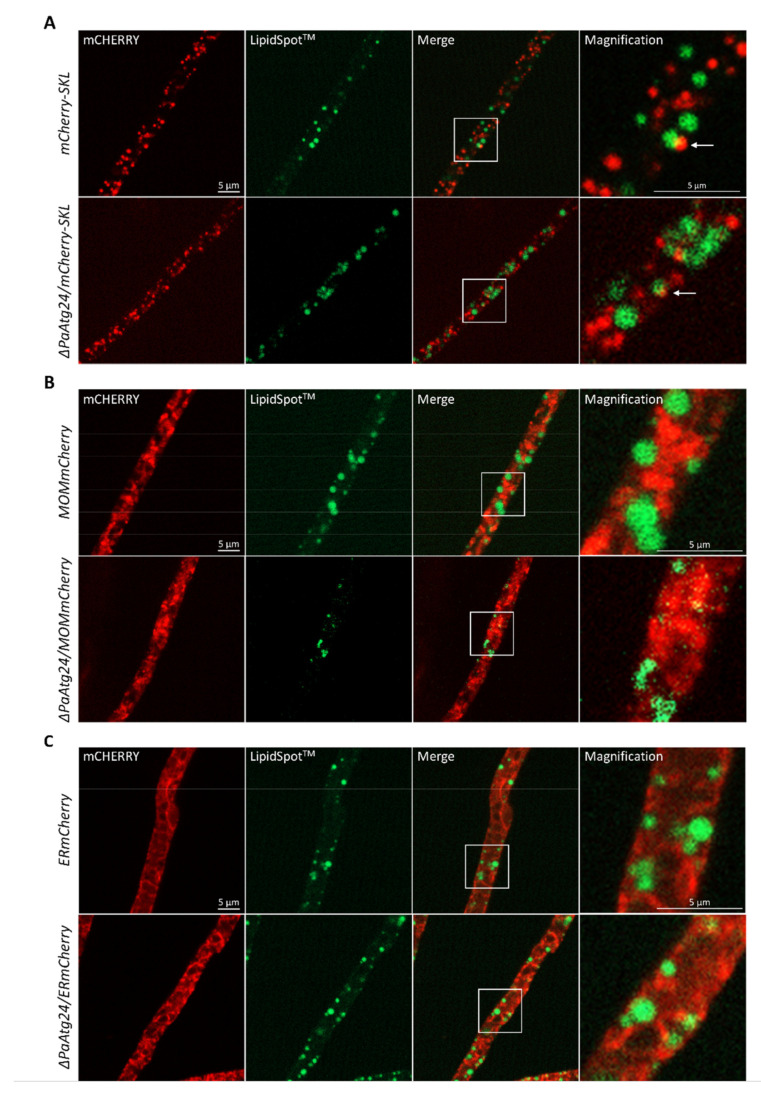
Lipid droplet contact sites with peroxisomes, mitochondria and ER. (**A**) Fluorescence microscopic analyses of 7-day-old *mCherry-SKL* and *ΔPaAtg24/mCherry-SKL* strains to visualize peroxisomes (**A**), *MOMmCherry* and *ΔPaAtg24/MOMmCherry* strains to display mitochondria (**B**) as well as *ERmCherry* and *ΔPaAtg24/ERmCherry* strains to observe ER (**C**). Each sample (n = 3) was grown on M2O and stained for 15 min with LipidSpot™ for the visualization of lipid droplets. Arrows point to peroxisome-lipid droplet contact sites.

In order to validate this assumption, we next investigated whether or not lipid droplets contact peroxisomes in fluorescence microscopic analysis. This was clearly observed in a fluorescence microscopic analysis of 7-day-old (Figure 9A) and 14-day-old strains (Figure S6). In addition, lipid droplets are described as playing an important role in the detoxification of the protein aggregates of damaged and misfolded proteins from the ER [40] as well as in supporting mitochondria during stress and aging via the incorporation of harmful proteins and lipids to prevent apoptosis and lipotoxicity [41]. Lipid droplets, which are loaded with these proteins and lipids, are subsequently eliminated via lipophagy, a form of selective autophagy. Previously, it was demonstrated that this autophagic process is a modulator of longevity in *C. elegans* [42]. To analyze whether or not lipid droplets contact mitochondria and ER in *P. anserina* as well, we performed further fluorescence microscopic analyses. Close contact between lipid droplets and mitochondria, as well as lipid droplets and the ER, was observed in 7-day-old cultures (Figure 9B,C) as well as 14-day-old cultures (Figure S6). We assume that the occurrence of lipid droplets and their interaction with ER, mitochondria and peroxisomes is beneficial in *P. anserina*, like in *C. elegans*, and contributes to the increased lifespan of both strains on M2O compared to M2.

**Figure 10:**
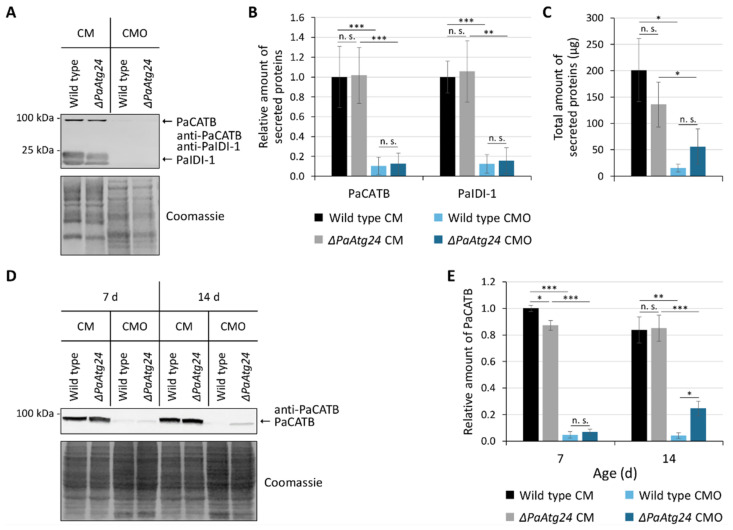
Down-regulation of protein secretion by oleic acid diet. (**A**) Western blot analysis with secreted protein samples from 7- and 14-day old wild type and *ΔPaAtg24* cultures grown in liquid CM or CMO medium. The membrane was treated with an antibody against secreted catalase PaCATB and cell wall protein PaIDI-1. (**B**) Quantification of PaCATB and PaIDI-1 protein amount from strains from (**A**) normalized to Coomassie stained gels. (**C**) The total amount of secreted proteins per strain in the growth medium (150 mL) from samples from (**A**) was measured spectrophotometrically with Bradford reagent. (**D**) Western blot analysis with total protein extract from 7- and 14-day-old wild type and *ΔPaAtg24*, grown in CM or CMO. The membrane was treated with an antibody against PaCATB. (**E**) Quantification of PaCATB protein amount from strains from (**D**) normalized to Coomassie stained gels. Data represent mean ± SD (n = 4). Samples were statistically analyzed with two-tailed *t*-test (n. s. = *p* > 0.05; * = *p* ≤ 0.05; ** = *p* ≤ 0.01; *** = *p* ≤ 0.001). For data point distribution, see Figure S7.

Lipid droplets transport oleic acid from the site of entrance to the peroxisomes. In contrast, numerous secretory vesicles are transported in the opposite direction to deliver secreted proteins to the hyphal tips. This type of growth is typical for filamentous fungi, such as *P. anserina* [43]. The two membrane-dependent processes compete with each other. In order to elucidate which process dominates in *P. anserina* grown on oleic acid medium, we isolated the liquid cultivation medium after growth of strains for 4 days and analyzed the medium for the presence of two known secreted proteins, catalase PaCATB and the cell wall protein PaIDI-1, via western blot analysis (Figure 10A). The amount of the two proteins was quantified (Figure 10B). In addition, the total amount of secreted proteins treated with Bradford reagent was determined spectrophotometrically (Figure 10C).

Under standard growth conditions, *P. anserina* secretes a range of proteins (Figure 10A–C). For this process, secretory vesicle transport to the plasma membrane is required. We found that in *ΔPaAtg24*, protein secretion is slightly decreased (Figure 10C). This decrease may be the reason for the reduced production of aerial hyphae and fruiting bodies of this strain [9], because secreted proteins (e.g., hydrophobines) are essential for these developmental processes [44]. Surprisingly, in CMO medium, the total amount of secreted proteins is strongly reduced (Figure 10C).

Next, we analyzed whether the secretion or protein amount of the secreted catalase B (PaCATB) is reduced. Therefore, we performed a western blot analysis with total protein extracts that uncovered a strong decrease in the amount of this protein (Figure 10D,E). We assume that the reduction of secretory protein trafficking is caused by the need for lipid droplets for intracellular oleate transport in CMO medium. Since, under the applied conditions, oleic acid is the sole carbon source and the metabolization of this fatty acid is essential for survival, *P. anserina* strains seem to adapt to these conditions by favoring oleate uptake and down-regulating protein secretion, probably via a reduction of the amount of secreted proteins like PaCATB to ensure proper nutrient supply.

### 3.6. Oleic Acid Diet Abolishes Mislocalization of SNARE PaSNC1 in ΔPaAtg24

In yeast, one function of sorting nexin ATG24 is the recycling of the v-SNARE SNC1 [8,45]. This protein is inserted into the membrane of secretory vesicles and mediates the fusion events of the corresponding vesicles with the plasma membrane. Subsequently, SNC1 recycles back to the Golgi apparatus. In contrast, in the absence of ATG24, this v-SNARE is missorted to the vacuole [46].

**Figure 11:**
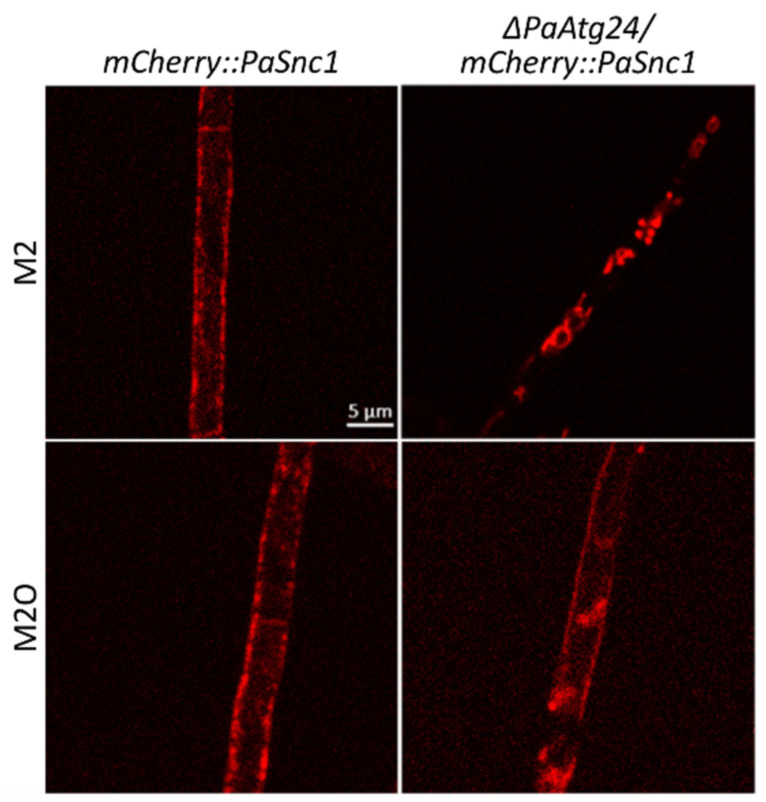
*ΔPaAtg24* shows a mislocalization of PaSNC1, which is neutralized by oleic acid diet. Fluorescence microscopic analysis of mCHERRY::PaSNC1 localization in 7-day-old *mCherry::PaSnc1* and *ΔPaAtg24/mCherry::PaSnc1* strains on M2 and M2O medium. For every sample, three biological replicates were analyzed.

In order to analyze whether or not PaATG24 is also necessary for the recycling of PaSNC1 in *P. anserina*, we generated two *P. anserina* strains in which the *mCherry::PaSnc1* fusion gene is expressed in the wild-type and *ΔPaAtg24* background, respectively (Figure S8). Fluorescence microscopy revealed that in the control strain grown on both M2 and M2O, mCHERRY-tagged PaSNC1 localizes to the plasma membrane (Figure 11). In the *ΔPaAtg24* strain, grown on M2 medium, PaSNC1 is found in the cytoplasm. The growth of this strain on oleic acid almost completely restores the plasma membrane localization of PaSNC1. These data suggest a role of PaATG24 in the recycling of PaSNC1 as it is described for the yeast homologue [46]. Moreover, in the absence of PaATG24, the oleic acid diet leads to a normalization of membrane trafficking (Figure 7).

### 3.7. Impact of Oleic Acid Diet on the Abundance and Morphology of Peroxisomes, Mitochondria and ER

**Figure 12:**
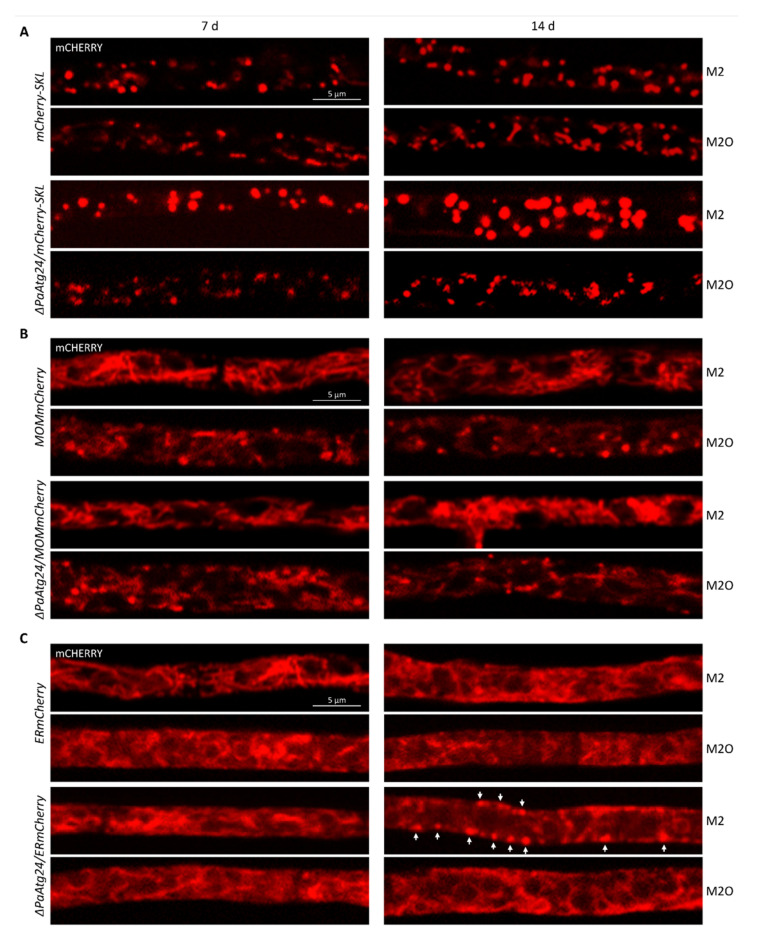
Peroxisome, mitochondria and ER morphology and abundance in dependence on oleic acid diet and PaATG24. (**A**–**C**) Fluorescence microscopic analyses of 7- and 14-day-old *mCherry* strains (n = 3), grown on M2 and M2O agar, respectively. Visualization of (**A**) peroxisomes was performed with *mCherry-SKL* and *ΔPaAtg24/mCherry-SKL* strains and mitochondria are displayed with *MOMmCherry* and *ΔPaAtg24/MOMmCherry* strains (**B**). For visualization of ER, *ERmCherry* and *ΔPaAtg24/ERmCherry* strains were analyzed (**C**). Arrows point to ER–plasma membrane contact sites.

Since *ΔPaAtg24* is impaired in mitophagy, pexophagy [9] and ER-phagy (Figure S5) under standard conditions, and since the oleic acid diet results in wild type-like autophagy flux (Figure 5), we next investigated the morphology and abundance of peroxisomes, mitochondria and ER under these conditions. Therefore, fluorescence microscopic experiments with reporter strains possessing mCHERRY-labelled organelles were performed under standard and oleic acid conditions, respectively. Under standard growth conditions, the absence of PaATG24 leads to an age-dependent increase of peroxisomal size (Figure 12A). A possible explanation for the enlargement of peroxisomes may be impaired pexophagy in this strain. Pexophagy is tightly linked to peroxisome fission [47,48], thus impairments in pexophagy might result in enlarged peroxisomes. To test this idea, we deleted the gene encoding the pexophagy regulator *PaAtg37*. These strains are deficient in pexophagy, but not in mitophagy, as expected, and also shows enlarged, rounded peroxisomes in old age (Figure S9). In addition, the peroxisome activity of *ΔPaAtg24* is strongly decreased (Figure 1A), underlining the importance of pexophagy as a peroxisomal quality control mechanism. Interestingly, the oleic acid diet neutralizes this effect and leads to wild-type size of peroxisomes in 14-day-old *ΔPaAtg24* (Figure 12A), which is linked to a wild-type autophagy rate in this strain (Figure 5).

Mitochondria show a filamentous morphology under standard conditions (Figure 12B). Compared to the wild type, the absence of PaATG24 leads to a slightly increased abundance of the mitochondria of 14-day-old cultures. This may be caused by the impairment of mitophagy under these conditions [9]. The oleic acid diet results in a slightly altered morphology. Both strains show more punctate mitochondria compared to standard medium. In addition, amounts of mitochondria in *ΔPaAtg24* are wild-type-like on M2O (Figure 12B).

In the absence of PaATG24, the morphology of the ER changes during aging. In 14-day-old strains, the ER is more punctate in comparison to the wild type and localizes to the plasma membrane (Figure 12C, marked with arrows). We speculate that these structures are ER–plasma membrane contact sites. These contacts play a role in the control of cellular Ca^2+^ homeostasis as well as the exchange of lipids (reviewed in [49]). In eukaryotic cells, the majority of membrane lipids are synthesized in the ER and delivered to other membranes from which lipid metabolites are returned to the ER for metabolic recycling (reviewed in [50]). One pathway of recycling is bulk lipid transport by vesicular traffic along the secretory and endocytic pathways; another pathway is the direct delivery of membrane lipids from the ER to the plasma membrane. The inhibition of vesicular transport by genetic or pharmacological manipulations does not affect the delivery of membrane lipids from the ER to the plasma membrane [49]. In yeast, it has been shown that ER-synthesized sterol is transported to the plasma membrane independently of intracellular vesicular trafficking events [51]. In mammalians, non-vesicular sterol transport mediated by ER-resident proteins has been described [52]. Since vesicle transport is impaired in *ΔPaAtg24*, the delivery of membrane lipids directly from the ER to the plasma membrane may still be possible, but requires increased numbers of ER–plasma membrane contact sites on M2, as visible in Figure 12C. This effect is neutralized by oleic acid, which causes the abrogation of the membrane trafficking defect of *ΔPaAtg24* (Figure 7).

Overall, *ΔPaAtg24* shows alterations in peroxisome and ER morphology in old age on M2, which are neutralized by the oleic acid diet. We assume that this neutralization is caused by restored autophagy and membrane trafficking (Figure 5 and Figure 7).

### 3.8. Model Explaining the Impact of Oleic Acid on Membrane Trafficking

**Figure 13:**
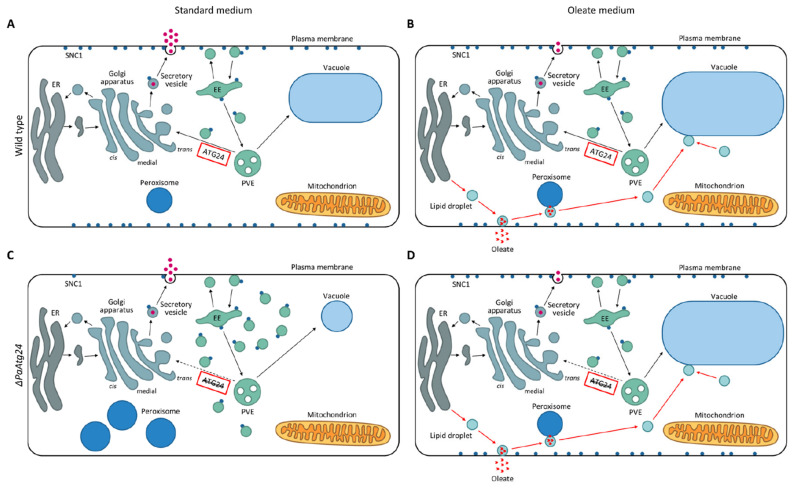
Model comparing the effects of an oleate diet on membrane trafficking, vacuole formation and peroxisome function in *P. anserina* wild type and the *PaAtg24* deletion mutant. Membrane trafficking in the wild type on standard medium (**A**) and oleate medium (**B**) as well as in *ΔPaAtg24* on standard medium (**C**) and oleate medium (**D**). The oleic acid diet leads to the formation of lipid droplets for oleic acid transport to peroxisomes for metabolization. Compared to standard conditions, protein secretion is down-regulated and vacuoles are larger (**B**,**D**). The absence of PaATG24 results in an accumulation of endosomes because of blocked recycling (**C**). The oleic acid diet is beneficial for *P. anserina*, since it leads to an altered membrane trafficking and restores vacuole size and endosomal sorting in *ΔPaAtg24* (**D**). See main text for details. Abbreviations: EE = early endosome; ER = endoplasmatic reticulum; PVE = prevacuolar endosome.

Applying an oleic acid diet on *P. anserina* is beneficial for the fungus. Oleate metabolization results in a reduced ROS production, since it affects respiratory complex usage with bypassing complex I and II leading to an elongated lifespan of the wild type. In addition, it alters membrane trafficking, which allows short-lived *ΔPaAtg24* to increase lifespan as well. The absence of sorting nexin PaATG24 results in a mislocalization of SNARE PaSNC1, because PaATG24 is necessary for the recycling of this protein. SNC1 is located on vesicles and plays a role for vesicle–membrane fusion events. The deletion of *PaAtg24* leads to an accumulation of endosomes in the cytosol, highlighting the importance of PaATG24 for membrane sorting. Consequently, the available membranes are limited and vacuolar size is strongly reduced (Figure 13). Thus, the mutant is impaired in different forms of autophagy and produces increased amounts of ROS.

The oleic acid diet is able to neutralize the described effects. This is linked to altered membrane trafficking. Secretion pathways are down-regulated, suggesting that membranes are used for lipid droplets instead of secretory vesicles, since lipid droplets are used for oleate uptake and its transport to the peroxisomes for metabolization. In yeast, a consumption of lipid droplets by the vacuole for membrane proliferation is described [53,54]. We assume that in *P. anserina*, lipid droplets also fuse with the vacuole, which subsequently results in the observed increase in vacuolar size compared to standard medium in wild type and *ΔPaAtg24* (Figure 13). This gain in vacuolar capacity results in a suspension of the autophagy defect of *ΔPaAtg24*, a wild type-like autophagy rate, a strong reduction of ROS generation, and an elongated lifespan. Overall, our data emphasize the relevance of nutritional cues for aging and health by the usage of an altered metabolism and controlling different pathways such as autophagy and membrane trafficking.

## 4. Conclusions

In this study, we reported an effect of an oleic acid diet on the lifespan of *P. anserina*. This effect is linked to alterations of the mitochondrial electron transport, ROS production, membrane trafficking, the formation of vacuoles and on autophagy. It is now a question of how far these effects, or parts of them, are conserved among organisms or whether they are specific for *P. anserina*. In this respect, it is interesting to consider that a Mediterranean diet in humans is highly beneficial for human health and aging [55]. This diet is dominated by the consumption of olive oil, which is rich in oleic acid. For instance, numerous studies suggest a positive effect of olive oil/oleic acid on cancer, autoimmune and inflammatory diseases as well as wound healing [56]. Furthermore, an antioxidant potential is described, which is protective against age-related forms of molecular damage [57]. A positive effect of oleic acid on the lifespan of *C. elegans* worms has also been described [58]. It will now be interesting to see whether or not the effects identified in our study can be translated to other biological systems, including the human species.

## Data Availability

Original data are available upon reasonable request from the corresponding author.

## Supplementary Materials

The following are available online at https://www.mdpi.com/article/10.3390/cells11030519/s1, Figure S1: Oleic acid diet rescues peroxisomal functionality of *ΔPaAtg24*; Figure S2: Oleic acid diet bypasses complex I and II of the mitochondrial respiratory chain; Figure S3: Metabolization of oleic acid results in lower hydrogen peroxide production; Figure S4: Abrogation of the *ΔPaAtg24* autophagy defect by oleic acid diet; Figure S5: *ΔPaAtg24* is impaired in ER-phagy; Figure S6: Lipid droplet contact sites with peroxisomes, mitochondria and ER; Figure S7: Down-regulation of protein secretion by oleic acid diet; Figure S8: Verification of newly generated *ERmCherry*, *ΔPaAtg24/ERmCherry*, *MOMmCherry*, *ΔPaAtg24/MOMmCherry*, *mCherry::PaSnc1* and *ΔPaAtg24/mCherry::PaSnc1* strains by Southern blot analyses; Figure S9: *ΔPaAtg37* is pexophagy-deficient.
