# Supplementary material for: Lifespan Increase of Podospora anserina by Oleic Acid Is Linked to Alterations in Energy Metabolism, Membrane Trafficking and Autophagy
Source: Cells. 2022 Feb 2;11(3):519. doi: 10.3390/cells11030519 (PMC8834509; doi:10.3390/cells11030519)
Supplement: Supplementary file 1 [file cells-11-00519-s001.zip › cells-1581461-supplementary.pdf]

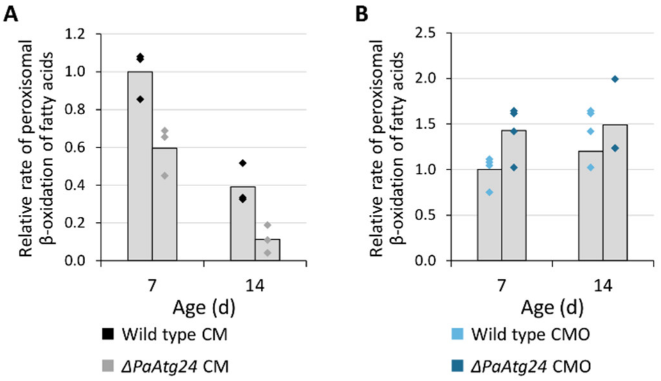

Figure S1. Oleic acid diet rescues peroxisomal functionality of *ΔPaAtg24*. Measurements of peroxisomal β-oxidation rate of fatty acids in 100 µg total protein extract from 7- and 14-day-old wild type and *ΔPaAtg24*, grown in (**A**) standard medium (CM) or (**B**) oleic acid-containing medium (CMO). Data represent mean and individual data points (n = 3). For statistical analysis see Figure 1.

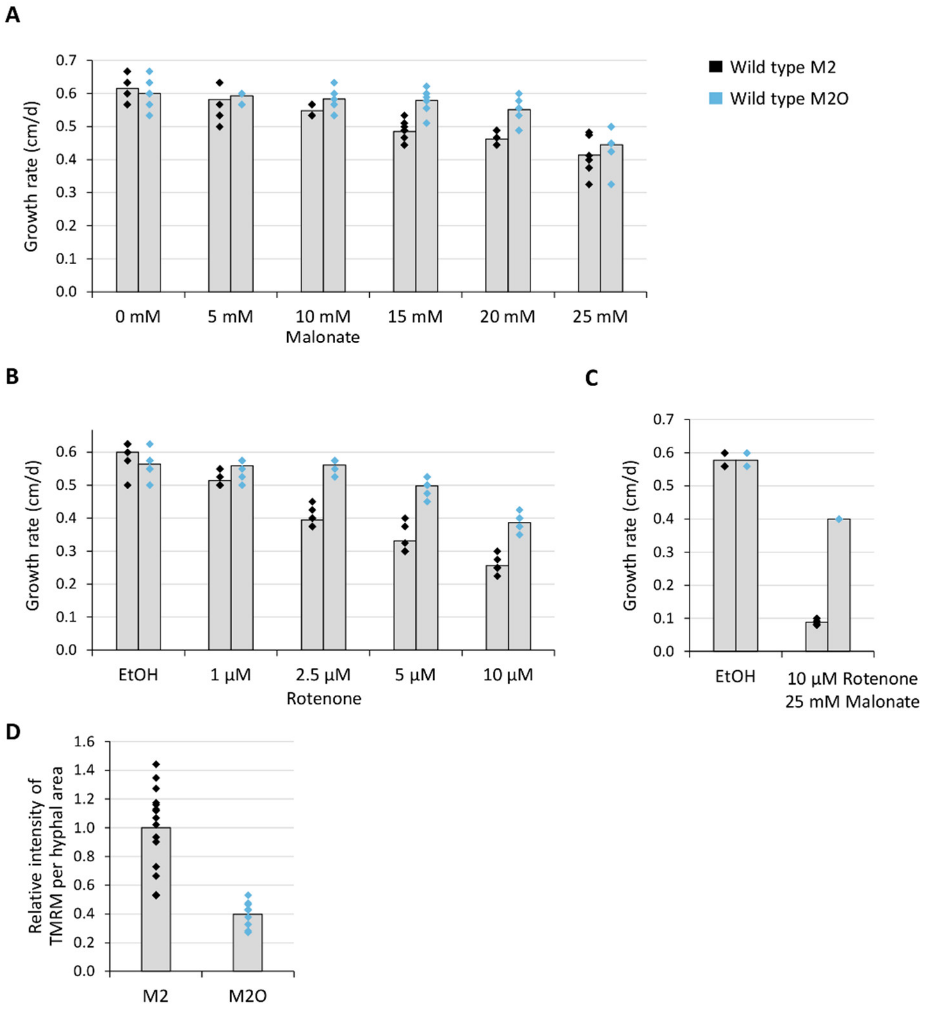

Figure S2. Oleic acid diet by-passes complex I and II of the mitochondrial respiratory chain. Growth tests of the wild type on standard medium (M2) and oleate-containing medium (M2O) with different concentration of complex II inhibitor malonate (**A**), complex I inhibitor rotenone (**B**) and a combination of both (**C**) were performed (n = 9). The relative intensity of TMRM signal per hyphal area is displayed in (**D**). 2498 (M2) or 2478 µm^2 (M2O) hyphae area was analyzed (n = 3). Data represent mean and individual data points. For statistical analysis see Figure 3.

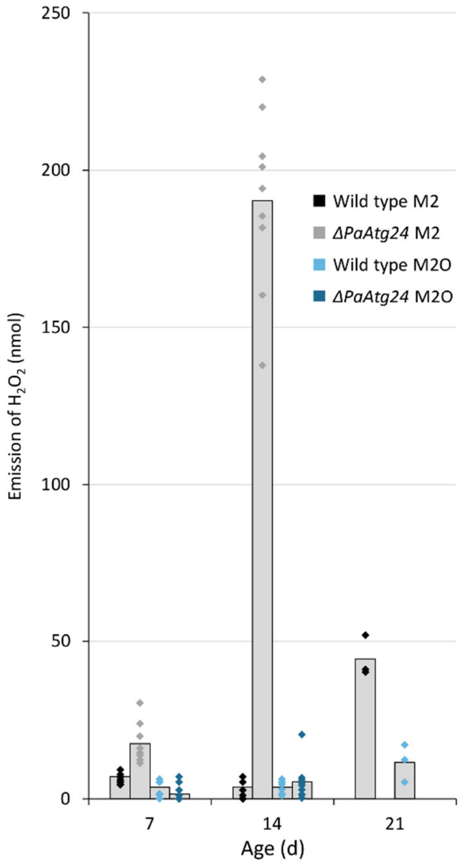

Figure S3. Metabolization of oleic acid results in lower hydrogen peroxide production. Quantitative measurement of H_2O_2 release with 7- and 14-day-old wild type and *ΔPaAtg24* on M2 and M2O medium (n = 9) as well as 21-day-old wild type (n = 3). Data represent mean and individual data points. For statistical analysis see Figure 4.

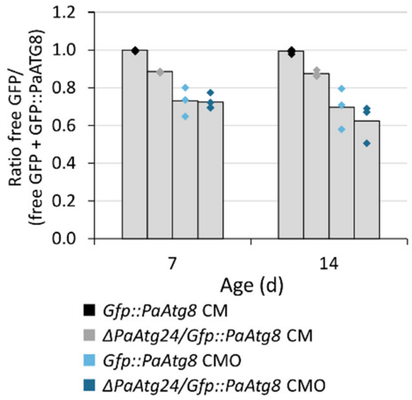

Figure S4. Abrogation of the *ΔPaAtg24* autophagy defect by oleic acid diet. Quantification of the ratio of free GFP to the total of free GFP and fusion protein from western blot analysis with total protein extracts from 7- and 14-day-old *Gfp::PaAtg8* and *ΔPaAtg24/Gfp::PaAtg8*, grown in liquid CM or CMO medium. The membrane was treated with an antibody against GFP. Data represent mean and individual data points (n = 4). For statistical analysis see Figure 5.

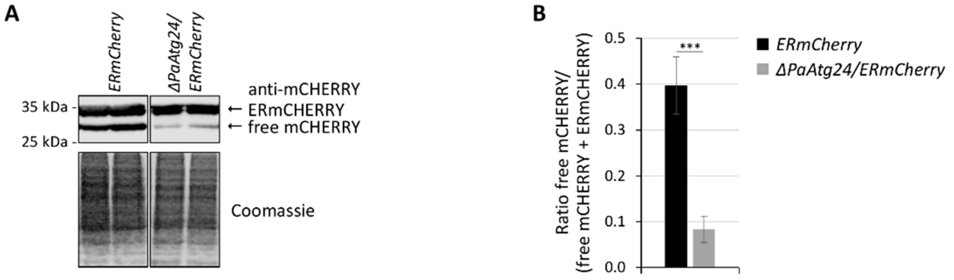

Figure S5. *ΔPaAtg24* is impaired in ER-phagy. (**A**) Western blot analysis of total protein extracts from 7-day-old *ERmCherry* and *ΔPaAtg24/ERmCherry* strains, grown under standard conditions. The membrane was treated with an antibody against mCHERRY. (**B**) Quantification of ratio of free mCHERRY to the total of free mCHERRY and fusion protein from samples from (**A**). Data represent mean ± SD (n = 4). Samples were statistically analyzed with two-tailed *t*-test (*** = $p \leq 0.001$).

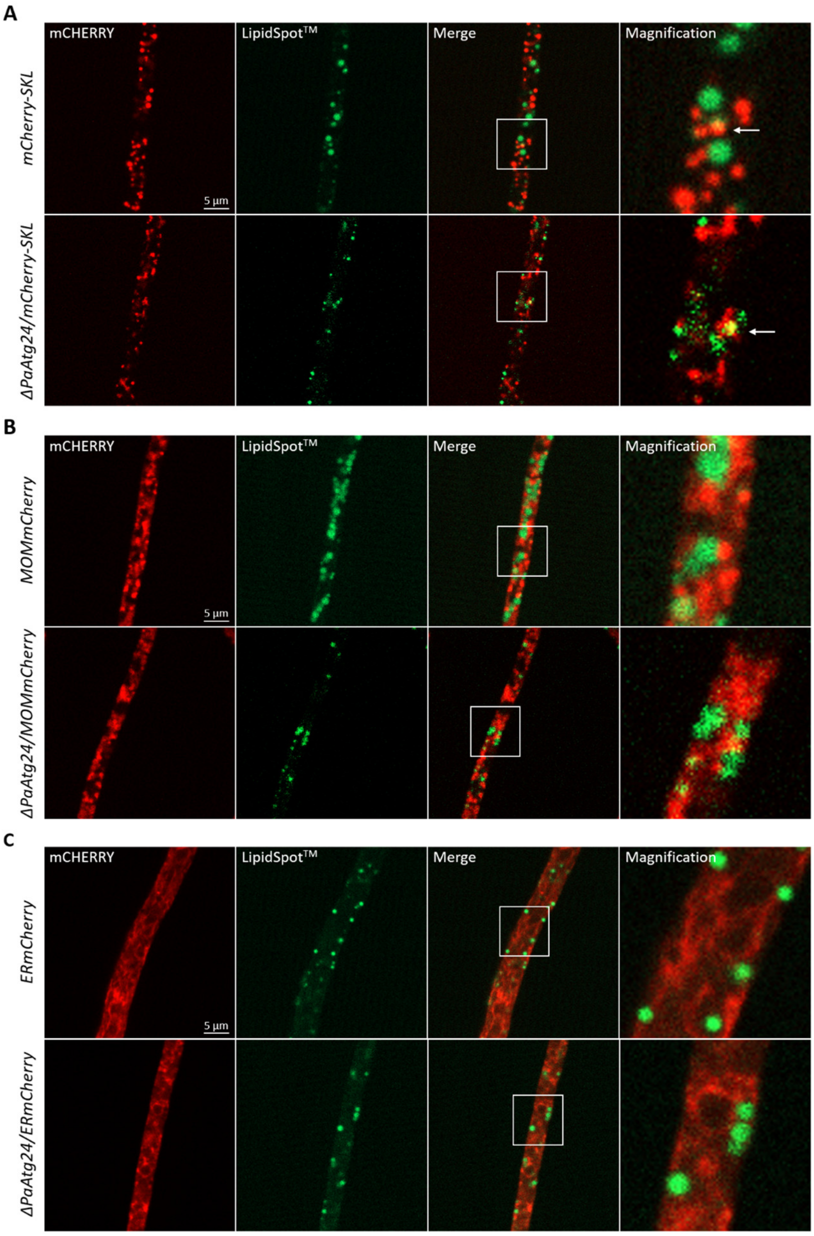

Figure S6. Lipid droplet contact sites with peroxisomes, mitochondria and ER. (**A**) Fluorescence microscopic analyses of 14-day-old *mCherry-SKL* and *ΔPaAtg24/mCherry-SKL* strains to visualize peroxisomes (**A**), *MOMmCherry* and *ΔPaAtg24/MOMmCherry* strains to display mitochondria (**B**) as

well as *ERmCherry* and *ΔPaAtg24/ERmCherry* strains to observe ER (**C**). Each sample (n = 3) was grown on M2O and stained for 15 min with LipidSpot™ for visualization of lipid droplets. Arrows point to peroxisome-lipid droplet contact sites.

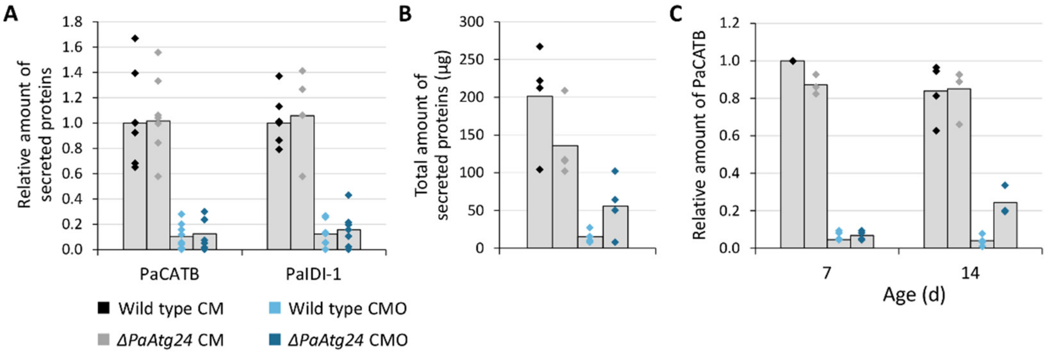

Figure S7. Down-regulation of protein secretion by oleic acid diet. (**A**) Quantification of PaCATB and PaIDI-1 protein amount (normalized to Coomassie stained gels) from western blot analysis with secreted protein samples from 7- and 14-day old wild type and *ΔPaAtg24* cultures grown in liquid CM or CMO medium. (**B**) The total amount of secreted proteins per strain in the growth medium (150 mL) from samples from (**A**) was measured spectrophotometrically with Bradford reagent. (**C**) Quantification of PaCATB protein amount (normalized to Coomassie stained gels) from western blot analysis with total protein extract from 7- and 14-day-old wild type and *ΔPaAtg24*, grown in CM or CMO. Data represent mean and individual data points (n = 4). For statistical analysis see Figure 10.

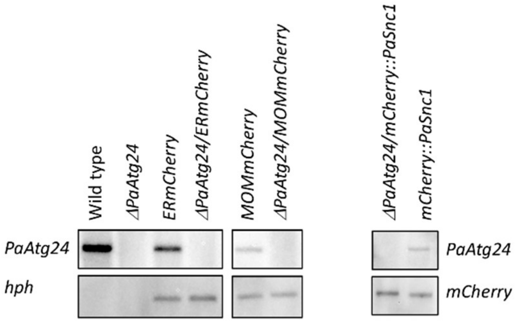

Figure S8. Verification of newly generated *ERmCherry*, *ΔPaAtg24/ERmCherry*, *MOMmCherry*, *ΔPaAtg24/MOMmCherry*, *mCherry::PaSnc1* and *ΔPaAtg24/mCherry::PaSnc1* strains by Southern blot analyses. 1500 ng genomic DNA was digested with enzyme EcoRV. *PaAtg24*-, *mCherry*- and *hph*-specific probes were used for detection.

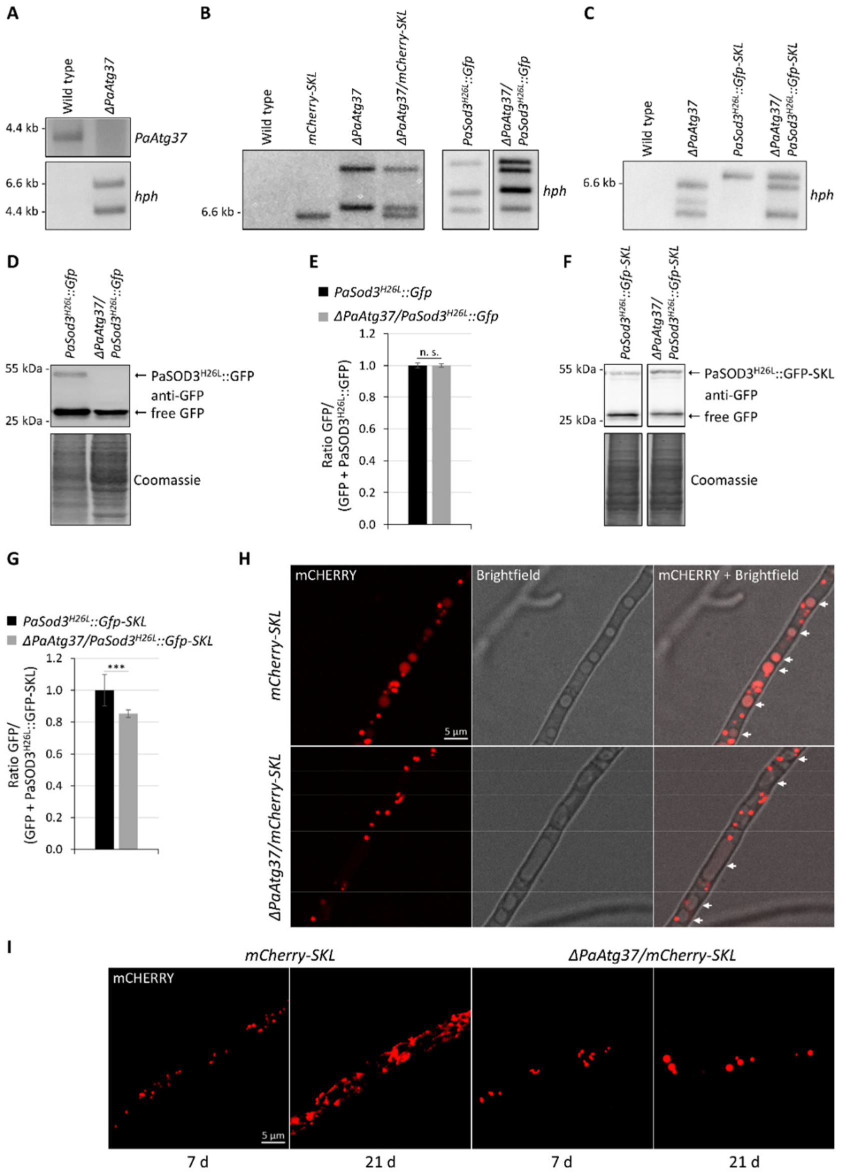

Figure S9. *ΔPaAtg37* is pexophagy-deficient. (**A**) Verification of the newly generated *ΔPaAtg37* strain by Southern blot analyses. 900 ng genomic DNA was digested with enzyme HindIII. *PaAtg37*- and *hph*-specific probes were used for detection. (**B**) Verification of newly generated *ΔPaAtg37/mCherry-SKL* and *ΔPaAtg37/PaSod3*^{H26L}*::Gfp* strains. 700 ng genomic DNA was digested with enzyme HindIII. An *hph*-specific probe was used for detection. (**C**) Verification of a newly generated *ΔPaAtg37/PaSod3*^{H26L}*::Gfp-SKL* strain. 800 ng genomic DNA was digested with enzyme EcoRV. An *hph*-specific probe was used for detection. (**D**) Western blot analysis of total protein extracts from 7-day-old *PaSod3*^{H26L}*::Gfp* and *ΔPaAtg37/PaSod3*^{H26L}*::Gfp* strains, grown under standard conditions, to measure mitophagy. The membrane was treated with an antibody against GFP. (**E**) Quantification of ratio of free GFP to the total of free GFP and fusion protein from samples from (**D**). (**F**) Western blot analysis of total protein extracts from 7-day-old *PaSod3*^{H26L}*::Gfp-SKL* and *ΔPaAtg37/PaSod3*^{H26L}*::Gfp-SKL* strains, grown under standard conditions, to measure pexophagy. The membrane was treated with an antibody against GFP. (**G**) Quantification of the ratio of free GFP to the total of free GFP and fusion protein from samples from (**F**). (**H**) Fluorescence microscopic analyses of 7-day-old *mCherry-SKL* and *ΔPaAtg37/mCherry-SKL* strains, cultivated on glass slides with M2 for 2 d, to display pexophagy in dependence of PaATG37. mCHERRY-stained vacuoles (*mCherry-SKL*) or "empty" vacuoles (*ΔPaAtg37/mCherry-SKL*) are marked with arrows. (**I**) Fluorescence microscopic analyses of 7- and 21-day-old *mCherry-SKL* and *ΔPaAtg37/mCherry-SKL*, cultivated on glass slides with M2 for 1 d to visualize peroxisomes. Data represent mean ± SD (n = 3). Samples were statistically analyzed with two-tailed *t*-test (n. s. = $p > 0.05$; *** = $p \leq 0.001$).
